# Contents of Functionally Bioactive Peptides, Free Amino Acids, and Biogenic Amines in Dutch-Type Cheese Models Produced with Different Lactobacilli

**DOI:** 10.3390/molecules25225465

**Published:** 2020-11-22

**Authors:** Monika Garbowska, Antoni Pluta, Anna Berthold-Pluta

**Affiliations:** Division of Milk Technology, Department of Food Technology and Assessment, Institute of Food Sciences, Warsaw University of Life Sciences-SGGW, Nowoursynowska 159c Street, 02-776 Warsaw, Poland; antoni_pluta@sggw.edu.pl (A.P.); anna_berthold@sggw.edu.pl (A.B.-P.)

**Keywords:** biogenic amines, free amino acids, *Lactobacillus*, L-carnosine, anserine, additional cultures

## Abstract

Cheese ripening involves a number of biochemical processes, mainly of a proteolytic nature, which are initially triggered principally by milk-coagulating enzymes and, afterward, by microorganisms or enzymes of microbial origin. The proteolytic reactions affect, primarily, the synthesis of macro- and medium-molecular peptides from casein. In turn, the advanced proteolysis ends in the formation of short peptides and free amino acids. Further reactions may lead to the formation of nutritionally unfavorable biogenic amines. The present study aimed to determine changes in the contents of bioactive peptides (anserine and L-carnosine), free amino acids, and biogenic amines throughout the ripening of cheese models produced with the addition of *Lactobacillus* genus bacteria. The contents of amino acids varied considerably in the cheese models, depending on the bacterial strain added and ripening time. After five weeks of ripening, the total content of free amino acids in the cheese models ranged from 611.02 (a cheese model with *Lactobacillus casei* 2639) to 1596.64 mg kg^−1^ (a cheese model with *Lb. acidophilus* 2499). After the same time, the contents of the total biogenic amines in the cheese models with the addition of lactobacilli were lower than in the control cheese model (except for the model with *Lb. rhamnosus* 489). Anserine was detected in all cheese models (79.29–119.02 mg kg^−1^), whereas no L-carnosine was found over a five-week ripening period in the cheese models with *Lb. delbrueckii* 490 and *Lb. casei* 2639. After a five-week ripening, the highest total content of bioactive peptides was determined in the cheese models containing *Lb. acidophilus* 2499 (136.11 mg kg^−1^).

## 1. Introduction

Cheese ripening is a complex process entailing a series of biochemical reactions that are initially triggered mainly by milk-coagulating enzymes and, afterward, by microorganisms or enzymes derived from various sources. During ripening, the proteolysis is accompanied by the metabolic transformation of residual lactose, lactate, and citrate, and also, by lipolysis. Lactose fermentation to lactic acid is induced by viable microorganisms, including mainly these from starter cultures and accidental bacteria, especially in the case of artisan cheeses. The lipolysis process has been found relatively limited in most varieties of ripening cheeses having high contents of lactic acid bacteria, like Gouda, Cheddar, and Swiss types, while extensive in the cheese varieties ripening with molds or in the long-ripening ones (e.g., Italian and Swiss cheeses). In turn, proteolysis observed during cheese ripening is determined by endogenous proteolytic enzymes of milk (plasmin, somatic cell proteases, and cathepsins); exogenous proteases; and bacterial peptidases; and also, preparations of coagulating enzymes (mainly chymosin) and additional proteolytic enzymes of microbiological origin added during the technological process to accelerate the ripening process [1].

The proteolytic reactions induced by the activities of proteinases or peptidases during the ripening process affect, primarily, the synthesis of macro- and medium-molecular peptides from casein. The peptides formed in this way lead to severe textural modifications of cheese at the early stage of its ripening. Besides, large peptides and oligopeptides contribute to the development of a beneficial (desired/typical) taste of cheese, acting mainly as substrates for the enzymatic microbiota [2]. In turn, the advanced proteolysis ends in the formation of short peptides and free amino acids, which have a great impact on cheese aroma development [1]. Lactic acid bacteria (LAB) contribute to the synthesis of various substances during milk protein proteolysis that may positively affect a human body, like, e.g., amino acids, including ornithine, which exhibits sedating, soporific, and fatigue-attenuating effects [3,4]. Peptides encoded in the structures of precursor proteins activated via the proteolysis of the inactive forms of these proteins are called bioactive peptides. They are usually represented by short-chain peptides built of 2–20 amino acid residues [5] and perform various physiological functions, including antihypertensive, anticoagulant, antimicrobial, antioxidant, opioid, metal-binding, microelement-binding, and anticarcinogenic ones [6].

Cheeses may be perceived as an essential source of bioactive peptides due to a high protein content and a variety of proteolytic enzymes, as well as the extent and magnitude of proteolysis ongoing during cheese ripening. Numerous bioactive peptides formed upon proteolysis can be successively hydrolyzed to new short-chain peptides in more advanced proteolytic reactions [7]. Hence, cheeses may contain multiple types of peptides serving various biofunctions, depending on their ripening degree. Many scientists have investigated the presence and functional properties of various bioactive peptides formed during cheese ripening [8,9,10]. Positive properties have been ascribed to such bioactive peptides as L-carnosine (β-alanyl histidine) and anserine (1-methyl carnosine). They can be found in the skeletal muscles and nervous tissues of fish, birds, and mammals. In a human body, their contents are determined by gender, age, and diet, whereas their synthesis in skeletal muscles by the availability of alanine and histidine [11]. Research results suggest that diet supplementation with carnosine can be effective in the prevention or treatment of neurodegenerative diseases (e.g., Parkinson’s and Alzheimer’s diseases). Furthermore, L-carnosine exhibits metal ion-chelating properties (including ions of iron, copper, zinc, and cobalt) and, also, antiglycating, antioxidative, and anti-crosslinking effects [11,12,13]. It is also considered as an aging-preventive endogenous neuroprotective agent. It has also been implicated in regulating blood pressure, exhibiting antiproliferative properties, and affecting cardiovascular system functions [11,14,15].

Since the biochemical processes proceeding during cheese ripening affect its taste, texture, and appearance, and, also, the synthesis of bioactive peptides, their in-depth investigation seems essential to control and modify the course of the ripening process. This knowledge would be indispensable while, e.g., selecting appropriate bacterial cultures used to produce ripening cheeses [1].

**Figure 1:**
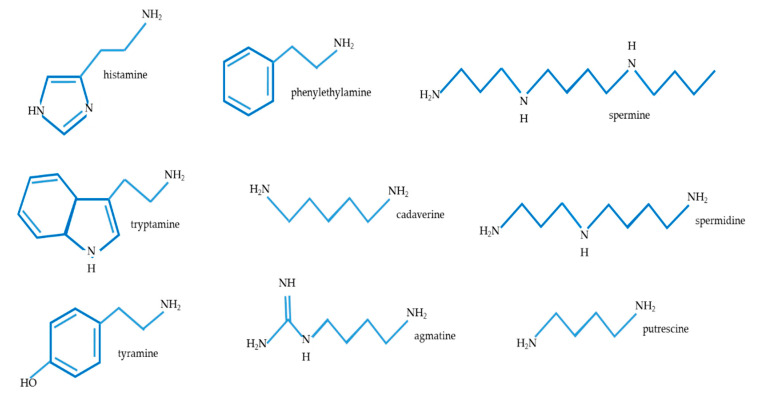
The molecular structures of common amines.

The free amino acids synthesized upon proteolysis also include amino acids that can undergo decarboxylation, leading to the formation of biogenic amines (BAs), whose accumulation in food products is undesirable [16,17,18,19]. Biogenic amines are organic nitrogen compounds, having low molecular weights and displaying an aliphatic (putrescine, cadaverine, spermine, or spermidine); aromatic (tyramine or phenylethylamine); or heterocyclic structure (histamine or tryptamine) (Figure 1). They are synthesized to the respective free precursor amino acids via decarboxylation and via the catalytic action of specific bacterial decarboxylases. For example, histamine is a bioactive amine that is synthesized by decarboxylation of its precursor amino acid, histidine, in an enzymatic reaction involving L-histidine decarboxylase. Tyramine, putrescine, cadaverine, and 2-phenylethylamine are products of the decarboxylation of tyrosine, ornithine (and/or agmatine) lysine, and phenylalanine, respectively [17,19,20].

Literature works report on BAs presence in different cheese varieties produced from cow, sheep, and goat milks [21,22,23]. Higher contents of BAs are usually found in cheeses made of raw than pasteurized milk. BAs synthesis in cheeses is also significantly affected by the presence of decarboxylating microorganisms [24], including, mainly, *Enterococcus* and *Lactobacillus*; however, the enterococci are prevailing bacteria in the long-ripening cheeses made of raw milk [25,26]. One of the most common BAs found in cheeses is tyramine, which contributes to migraines, increased cardiac output, nausea, vomiting, breathing disorders, and an increased blood glucose level in sensitive persons [27,28].

The analysis of BAs presence in food products seems interesting considering the toxicological risk they pose (e.g., the intake of histamine-rich food can adversely affect consumer health) [29] and their utilization as food quality indicators (e.g., for the determination of freshness or spoilage of food products) [30]. The toxic effects of BAs found in food include multiple adverse outcomes appearing in sensitive persons, like, e.g., headaches and dizziness, nausea and vomiting, hypotension or hypertension, palpitations, intracerebral hemorrhage, and anaphylactic shock syndrome [17,31]. Various strategies have been proposed to minimize BAs accumulation in cheeses, including the reduction of BA-producing microflora by the pasteurization of cheese milk, the use of starter cultures with lower BA-producing and proteolytic activities (thereby limiting the availability of BAs precursor amino acids), and by shortening the ripening process. However, in some cases, the characteristics of fermented foods make it difficult to implement these solutions [32].

Microorganisms exhibit various capabilities to produce biogenic amines and bioactive substances in the real environment of food products, including cheeses, which are strongly affected by this environment’s conditions (e.g., NaCl and pH). Therefore, it seems necessary to determine the capability of microorganisms to produce biogenic amines and bioactive substances not only in vitro but, also, in the real environment of food products. For this reason, a study was undertaken to determine the free amino acid composition, contents of bioactive dipeptides (L-carnosine and anserine), and contents of biogenic amines in cheese models produced with the addition of various *Lactobacillus* genus bacteria.

## 2. Results and Discussion

### 2.1. Contents of Free Amino Acids in Cheese Models Examined

The contents of individual free amino acids (FAAs) and total free amino acids (TFAAs) in Dutch-type cheese models manufactured with different cultures during ripening are shown in Table 1. The FAAs and TFAAs contents were significantly (*p* < 0.05) affected by the additional lactobacilli culture. No threonine or cystine was detected in any of the cheese variants throughout their five-week ripening. The cheese models with the additional *Lactobacillus acidophilus* 2499 culture had a high content of amino acids throughout the ripening period. After completed ripening, the highest contents were determined for asparagine, glutamic acid, and lysine. The histidine content decreased three-fold from 125 mg kg^−1^ in the first week of a ripening to 43.02 mg kg^−1^ after five weeks of ripening, whereas that of lysine increased four-fold, reaching 137.26 mg kg^−1^ within the same time span. In the cheese models with *Lb. acidophilus* 2499, the greatest fluctuations were observed for the glutamic acid content, which reached 56.16 mg kg^−1^ after one week, 235.09 mg kg^−1^ after three weeks, and 571.11 mg kg^−1^ after five weeks of ripening. The cheese models with *Lb. acidophilus* had the highest content of glutamic acid determined after a five-week ripening period compared to the other cheese models. This may be deemed a positive trait, because glutamic acid is responsible for cheese taste enhancement [33]. The cheese models produced with *Lb. acidophilus* 2499 culture showed a high TFAAs content as early as after the first week of ripening, when it reached 1083.51 mg kg^−1^. In the third and fifth weeks of ripening, the TFAAs content increased in this cheese variant to 1152.98 mg kg^−1^ and 1596.64 mg kg^−1^, respectively. After five weeks of ripening, the TFAAs content was the highest in this cheese model compared to the other models and almost equally high in the cheese models with the additional *Lb. rhamnosus* 489 culture. Asparagine, aspartic acid, glutamic acid, and valine were the major amino acids founds in the cheese models with *Lb. rhamnosus* 489, with their contents reaching 129.17, 119.89, 443.94, and 145.05 mg kg^−1^ after five weeks of ripening, respectively. After completing ripening, the cheese models with the *Lb. rhamnosus* culture had also the highest content of proline (96.57 mg kg^−1^), which can positively affect the flavor values of cheeses as it imparts a sweet taste to them [34]. In addition, after a five-week ripening, the cheese models with *Lb. rhamnosus* addition had the highest content of ornithine among all variants tested, which seems interesting considering its documented sedative properties [35]. Within the five-week ripening, the greatest increase in TFAAs content was noted in the cheese models with *Lb. rhamnosus* 489, i.e., from 595.99 mg kg^−1^ after the first week through 1255.57 mg kg^−1^ after the third week to 1555.83 mg kg^−1^ after the fifth week of ripening.

The cheese models produced with the adjunct *Lb. delbrueckii* 490 culture were characterized by a slightly lower content of amino acids than the two cheese model variants described above. They showed the highest histidine content after a five-week ripening compared to the other variants examined. A histidine-rich diet has been proven to increase the carnosine level in the skeletal muscles of humans [11]. The total content of free amino acids in these cheese models reached 775.69 mg kg^−1^ after one week, 881.57 mg kg^−1^ after three weeks, and 1142.14 mg kg^−1^ after five weeks of ripening.

In turn, the cheese models with the *Lb. casei* 2639 culture addition had the lowest TFAAs content after a five-week ripening (611.02 mg kg^−1^) among all cheese model variants, including the control one. None of the amino acids were found in these cheese models at ≥100 mg kg^−1^. After one week and three weeks of ripening, the TFAAs contents in these cheese models reached 409.91 and 520.53 mg kg^−1^, respectively, and did not differ significantly from that determined in the control cheese models.

The cheese models produced with the addition of *Lb. acidophilus*, *Lb. rhamnosus*, and *Lb. delbruckii* cultures had the highest contents of glutamic acid, which is converted by LAB occurring in fermented dairy drinks to Gamma-aminobutyric acid (GABA) via the glutamate decarboxylase-catalyzed decarboxylation [36]. GABA is an inhibitory neurotransmitter in the central nervous system of mammals synthesized in the body during sleep. Presumably, it is involved in sleep quality improvement after the intake of fermented dairy beverages [37,38]. The high content of glutamic acid in the cheese models tested can be deemed positive due to the possibility of its conversion by LAB to GABA and to the potential sleep quality improvement in consumers of food of this type.

The FAAs content of cheese is significantly determined by the proteolytic activity of microorganisms and, also, by their enzymatic system. The proteolytic enzymes of microflora are responsible for cleaving the terminal peptide bonds in proteins and peptides, which results in FAAs release. The subsequent conversions of FAAs and the synthesis of other products, like sensorially active substances or biogenic amines, are important as well [39]. The FAAs compositions in many cheese varieties have been addressed in ample research, and their contents have been found dependent on the cheese-making technology (including, type of curd, additional proteinases, starters, and ripening conditions); ripening time; and the extent and course of proteolysis [3,18,23,40].

### 2.2. The Presence of Biogenic Amines in Cheese Models Examined

The presence of BAs in cheeses has been well-documented so far and attributed mainly to the activity of nonstarter lactic acid bacteria (NSLAB), despite the intermediate role of the LAB’s proteolytic activity [41,42].

Lactobacilli are capable of relatively high BAs production in various fermented food products, including cheeses [43]. Even though various microorganisms (including Gram-negative bacteria) are able to synthesize BAs in cheeses, these are the *Lactobacillus* species, like *Lb. helveticus*, *Lb. buchneri*, and *Lb. curvatus*, that are claimed responsible for their accumulation in these food products [25,44,45]. The contents of BAs and total biogenic amines (TBAs) determined over the ripening period of Dutch-type cheese models manufactured with different lactobacilli are shown in Table 2. After five weeks of ripening, the TBAs contents ranged from 48.45 mg kg^−1^ in the cheese model with *Lb. casei* to 77.41 mg kg^−1^ in the cheese model with the additional *Lb. rhamnosus* culture. The differences observed in BAs contents between the cheese models examined were mainly due to their ripening time and the adjunct LAB culture used in the cheese-making process, which could promote BAs synthesis [19]. Pachlová et al. [40] determined the BAs contents in cheese models containing *Lb. curvatus* subsp. *curvatus* and *Lb. paracasei* strains capable of tyramine synthesis up to 200 mg kg^−1^ over the 90-day ripening period. In turn, Yilmaz and Görkmen [46] demonstrated the tyramine-producing ability of *Lb. plantarum* in yogurts and emphasized the potential intermediate effect of *Lb. delbrueckii* subsp. *bulgaricus* on tyramine accumulation in yogurts due to its synergistic interactions with tyraminogenic LAB. Among the cheese models examined in the present study, these with additional *Lb. delbrueckii* and *Lb. acidophilus* cultures had the highest TBAs contents after one week of ripening, i.e., 67.03 and 65.98 mg kg^−1^, respectively, which, however, decreased to 48.72 and 48.68 mg kg^−1^, respectively, after five weeks of ripening. No accumulation but a decrease in BAs contents after the five-week ripening of cheeses with lactobacilli additions can be explained by the presence of bacteria capable of BAs degradation, as confirmed by other authors [32,47,48]. Alvarez and Moreno-Arribas [47] suggested the use of BA-degrading strains isolated from various sources as an important alternative in reducing BAs contents in fermented food products. In turn, Herrero-Fresno et al. [32] found that the additional *Lactobacillus casei* 4a and 5b strains decreased BAs contents in models of Cabrales-type mini-cheese, even though they failed to identify the exact mechanism of this effect. The use of BA-degrading strains can be especially beneficial in cheese production from raw milk, in which both the nonstarter and nonlactic microflora is necessary to achieve the desired organoleptic traits of the finished product.

Over the five-week ripening period, no agmatine nor phenylethylamine were detected in any of the cheese models tested.

Over five weeks of ripening, the BAs contents increased in the control cheese models, these produced with additional *Lb. rhamnosus* and *Lb. casei* cultures, and decreased in these made with *Lb. delbrueckii* and *Lb. acidophilus* cultures. These changes can indicate that the two latter strains might be capable of BAs degradation, which can be considered beneficial considering their usability for producing cheese from raw milk.

In all cheese models analyzed in the present study, histamine (His) was found in the highest contents. Together with tyramine, it has been described as the most abundant biogenic amine of cheeses [18,49]. The cheese models produced with *Lb. rhamnosus* had a significantly (*p* < 0.05) higher His content after five weeks of ripening, compared to the other cheese variants. Food products that potentially contain the highest levels of histamine are ripened cheeses, in which the bacteria responsible for the fermentation and maturation have aminogenic capacity. Various lactic acid bacteria—also, those lactobacilli used in cheesemaking (e.g., *Lb. rhamnosus, Lb. casei, Lb. paracasei,* and *Lb. helveticus*)—have been described as histamine-producing microorganisms [50].

The contents of tyramine, putrescine, cadaverine, spermidine, and spermine determined throughout the ripening period were relatively low (<7 mg kg^−1^) in all cheese models. Both putrescine and cadaverine are responsible for the untypical taste of cheeses [47], and their considerable amounts are detected in overripened cheeses [51] and those made of raw milk [52]. The cadaverine presence in cheeses is due to the activity of contaminating bacteria, while its very low content is indicative of the good hygienic quality of milk used in the cheese-making process. It is also common knowledge that milk pasteurization contributes to the elimination of decarboxylating bacteria and, thereby, to the reduced BAs accumulation in dairy products [53]. The cheese models analyzed in the present study had low BAs contents mainly due to the proteolytic activity of the basic starter culture and the additional lactobacilli culture. In addition, BAs content determined after the five-week ripening of the cheese models was not high even despite their presence in cheeses noted in the first weeks of ripening. Therefore, after five weeks of ripening, no putrescine, spermine, and tyramine were detected in the cheese model with *Lb. rhamnosus*; no spermine and tyramine in the cheese model with *Lb. delbrueckii*; no putrescine, tryptamine, and tyramine in the cheese model with *Lb. acidophilus*; no tryptamine and tyramine in the cheese model with *Lb. casei*; and finally, no spermine and tryptamine in the control cheeses.

Histamine and tyramine are considered the most toxic and nonbeneficial from the food safety perspective. However, no toxicological level of BAs has been established in the European legislation. It is believed that BAs contents in foods at <100 mg/kg pose no severe risk to healthy consumers because they are metabolized in the intestines by detoxifying enzymes. In turn, higher BAs levels in foods can induce adverse psychoactive and vasoactive effects [21].

The use of additional bacterial cultures of *Lb. acidophilus* and *Lb. delbrueckii* in the present study contributed to a decrease in BAs content—histamine, in particular—in the cheese models studied.

### 2.3. The Presence of Anserine and Carnosine in Cheese Models Examined

The contents of individual bioactive peptides (BPs) and their total contents (TBPs) in cheese models are presented in Table 3. The TBPs contents were significantly (*p* < 0.05) affected by the additional culture and time of ripening. Like in the case of TFAAs contents, the lowest content of TBPs, reaching 136.11 mg kg^−1^, was determined after five weeks of ripening in the cheese models with the *Lb. acidophilus* 2499 culture addition. After a five-week ripening, these cheese models had also the highest contents of L-carnosine and anserine compared to the other cheese variants examined. The synthesis of L-carnosine in the skeletal muscles of humans depends on the availability of the following amino acids: alanine and histidine. After a five-week ripening, the highest content of alanine was determined in the cheese model with *Lb. acidophilus*, which might be due to the highest contents of L-carnosine and anserine in this cheese variant. Considering the above, this variant of cheese model can, apart from its basic nutritional value, offer additional health benefits.

No L-carnosine was detected in the cheese models with *Lb. delbrueckii* and *Lb. casei* cultures over their five-week ripening and in the control cheeses after three weeks of ripening. Our previous study [4], with cheese models produced with additional *Lactococcus* genus bacteria, demonstrated the levels of TBPs (anserine and carnosine) to range from 78.55 in most variants to 142.15 mg kg^−1^ in these with additional *Lactococcus lactis* 11454. The TBPs contents determined after a five-week ripening in the cheese models with additional lactobacilli cultures analyzed in the present study were slightly higher (80.64–136.11 mg kg^−1^) than in most variants of cheeses with *Lactococcus* strain additions.

The contents of anserine and carnosine determined in the analyzed cheese models were not high compared to these reported for other food products (0.24–7.11 mg g^−1^) [12,13]. However, given that carnosine and anserine contents are typically high in various meat and fish products due to a high carnosine content in the muscles, their lower contents were expected in the cheese models. Nevertheless, it is highly likely that carnosine plays a key biological role in the muscles and that its presence in various food products can prevent neurodegenerative diseases [12,13]. Therefore, the presence of bioactive peptides in cheeses at the end of their ripening period is, by far, beneficial. It also seems essential to determine the specific effects of the adjunct LAB cultures used in cheese-making.

## 3. Material and Methods

### 3.1. Experimental Material

The experimental material included five model cheeses produced with a CHN-19 (Chr. Hansen, Czosnów, Poland) culture (*L. lactis* ssp. *cremoris*, *L. mesenteroides* ssp. *cremoris*, and *L. lactis* ssp. *lactis biovar diacetylactis*) used as the basic starter and with *Lb. casei* 2639, *Lb. acidophilus* 2499, *Lb. rhamnosus* 489, and *Lb. delbrueckii* 49 (Polish Collection of Microorganisms of the Ludwik Hirszfeld Institute of Immunology and Experimental Therapy of the Polish Academy of Sciences in Wrocław, Poland).

### 3.2. Preparation of Cheese Models

Cheese models were prepared as described in our previous works [54,55].

### 3.3. Determination of Contents of Free Amino Acids, Biogenic Amines, and Bioactive Dipeptides in Cheese Models Using Liquid Chromatography Coupled with Electrospray Ionization Ion Trap Tandem Mass Spectrometry (LC-ESI-IT-MS/MS)

The contents of the free amino acids, biogenic amines, and bioactive dipeptides were determined in the cheese models using LC-ESI-IT-MS/MS following the methods described in our previous work [4]. Amino acids, biogenic amines, and bioactive peptides were determined with the Shimadzu LCMS 2020 HPLC system (Shim-Pol Inc., Warsaw, Poland) equipped with a DAD detector and a mass spectrometer operated in the ESI (electrospray ionization) mode. Separations were performed on a 5 mm × 2 mm × 250 mm COSMOSIL AR-II column thermostated at 85 °C. Mobile phase flow rate was 600 μL min^−1^. The following gradient was used for separations: Phase A—1% formic acid and B—20% methanol in acetonitrile: 0–15 min 9% B, 15–40 min 50% B, 40–50 min 80% B, and 50–60 min (column washing) 9% B. Mass spectrometer setup was as follows: DL temperature = 200 °C, nebulizing gas flow = 1 L min^−1^, heat block temperature = 350 °C, and drying gas flow = 15 L min^−1^.

### 3.4. Statistical Analysis

Results obtained were subjected to a statistical analysis using Statistica, version 13 software (TIBCO Software Inc., Kraków, Poland, 2017). One-way (ANOVA) analysis of variance was conducted. Tukey’s test was applied to compare the significance of the differences between the mean values at a significance level of α = 0.05.

## 4. Conclusions

The analyzed cheese models produced with the addition of lactobacilli revealed varied contents of TFAAs and FAAs over the five-week ripening period. Ornithine was detected in all variants of the cheese models after five weeks of their ripening, which might be found beneficial due to its documented bioactive functions. The cheese models examined had low contents of TBAs, mainly due to low histidine contents. The study showed that *Lb. acidophilus* and *Lb. delbrueckii* strains could be used as adjunct cultures capable of reducing the histamine contents, known as the most common and toxic BA in cheeses. In turn, the cheese models produced with the addition of *Lb. rhamnosus* 489 and *Lb. acidophilus* 2499 cultures had high contents of TFAAs (including ornithine) and TBPs (including L-carnosine), compared to the other cheese models tested. This can indicate their capability to synthesize bioactive substances during cheese ripening. To recapitulate, the choice of the *Lb. acidophilus* 2499 culture coupled with the use of a high-quality raw material—namely, milk—and appropriate technological treatments can offer a promising method for the manufacturing of food products posing a minimized health risk due to BAs presence and affecting the synthesis of substances with bioactive functions. The results obtained by us are the first in which it was found that anserine and L-carnosine occur in Dutch-type cheeses. The presence of these bioactive peptides may be related to the activity of the lactobacilli, which can be used as additional cultures in cheese production.

## Figures and Tables

**Table 1:** Concentration of individual and total free amino acids (TFAA) in Dutch-type cheese models during ripening (mg kg^−1^).

Added		Control			*Lb. rhamnosus* 489			*Lb. delbrueckii* 490			*Lb. acidophilus* 2499			*Lb. casei* 2639		
Amino Acids	Time of Ripening (Week)	1	3	5	1	3	5	1	3	5	1	3	5	1	3	5
Alanine		4.58 b ± 0.35	12.48 B ± 3.60	11.43 ^A^ ± 3.40	6.23 b ± 0.23	9.50 B ± 0.93	19.91 ^B^ ± 1.22	ND a	0.68 A ± 0.25	12.71 ^A^ ± 1.61	11.80 c ± 2.12	36.28 C ± 4.40	52.35 ^C^ ± 2.55	1.16 a ± 0.08	10.93 B ± 1.84	17.13^AB^ ± 1.44
Arginine		18.31 cd ± 2.40	17.68 A ± 3.28	16.59 ^A^ ± 3.34	ND a	16.51 A ± 0.56	16.81 ^A^ ± 1.24	20.86 d ± 1.28	18.33 A ± 1.12	16.28 ^A^ ± 0.38	15.56 bc ± 0.69	16.99 A ± 0.57	18.07 ^A^ ± 1.38	14.59 b ± 1.07	16.27 A ± 0.41	18.11 ^A^ ± 0.55
Asparagine		73.11 a ± 5.57	77.93 A ± 3.39	88.81 ^A^ ± 5.50	91.29 b ± 1.87	132.66 D ± 2.20	129.17 ^C^ ± 1.81	83.14 ab ± 7.38	95.16 B ± 3.90	108.74 ^B^ ± 10.12	75.53 ab ± 5.03	110.65 C ± 4.01	127.54 ^C^ ± 5.55	77.59 ab ± 9.69	79.68 A ± 4.85	84.16 ^A^ ± 5.21
Asparagine acid		16.56 a ± 0.39	31.45 B ± 3.58	54.50 ^C^ ± 5.38	72.03 d ± 1.96	113.21 D ± 4.00	119.89 ^E^ ±1.68	62.50 c ± 2.51	11.89 A ± 3.01	1.75 ^A^ ± 0.64	120.68 e ± 5.55	76.76 C ± 4.51	70.18 ^D^ ± 4.32	32.91 b ± 2.33	36.40 B ± 0.83	42.40 ^B^ ± 2.76
Glutamine		ND a	ND A	ND ^A^	20.22 b ± 3.59	41.21 D ± 1.59	38.71 ^C^ ± 2.25	ND a	13.34 B ± 0.34	12.74 ^B^ ± 0.42	ND a	24.88 C ± 3.54	34.47 ^C^ ± 4.85	ND a	ND A	ND ^A^
Glutamic acid		10.94 a ± 0.63	11.64 A ± 3.01	30.76 ^A^ ± 5.36	18.25 a ± 1.06	355.78 D ± 13.56	443.94 ^C^ ± 6.32	172.47 c ± 5.88	183.27 B ± 6.50	287.40 ^B^ ± 7.85	56.16 b ± 5.36	235.09 C ± 12.61	571.11 ^D^ ± 21.53	8.68 a ± 0.33	13.98 A ± 1.66	36.51 ^A^ ± 3.38
Glycine		0.30 a ± 0.09	0.32 A ± 0.18	0.49 ^A^ ± 0.19	5.04 b ± 0.12	14.06 C ± 1.61	14.26 ^C^ ± 2.46	0.36 a ± 0.05	3.85 B ± 0.77	1.97 ^A^ ± 0.59	0.30 a ± 0.27	3.37 B ± 0.50	6.00 ^B^ ± 0.22	0.14 a ± 0.01	1.05 A ± 0.06	1.30 ^A^ ± 0.01
Histidine		0.13 a ± 0.03	28.31 B ± 4.70	123.91 ^C^ ± 5.55	ND a	ND A	45.82 ^B^ ± 3.15	ND a	69.27 D ± 3.16	144.86 ^D^ ± 4.88	125.24 b ± 5.11	52.18 C ± 3.38	43.02 ^B^ ± 4.54	ND a	ND A	ND ^A^
Leucine		88.12 c ± 6.31	97.22 D ± 2.82	105.94 ^C^ ± 4.13	35.71 a ± 6.03	66.32 B ± 1.85	88.01 ^B^ ± 4.09	61.53 b ± 0.83	64.30 B ± 2.84	64.33 ^A^ ± 4.52	44.05 a ± 4.37	52.37 A ± 3.09	58.49 ^A^ ± 3.40	72.06 b ± 2.54	83.82 C ± 2.44	86.85 ^B^ ± 2.04
Isoleucine		84.40 d ± 5.58	77.09 C ± 6.79	70.88 ^B^ ± 4.08	55.42 b ± 3.04	67.22 BC ± 3.84	77.01 ^B^ ± 2.59	ND a	54.20 A ± 0.87	47.62 ^A^ ± 3.71	66.02 c ± 4.83	59.42 AB ± 4.35	52.80 ^A^ ± 2.71	66.61 c ± 1.51	75.81 C ± 5.71	79.42 ^B^ ± 3.02
Lysine		15.91 b ± 5.54	18.15 B ± 4.83	30.41 ^B^ ± 7.26	ND a	ND A	19.84 ^AB^ ± 2.05	24.63 c ± 0.16	29.38 C ± 6.13	129.15 ^C^ ± 5.12	29.85 c ± 2.27	40.54 D ± 3.36	137.26 ^C^ ± 5.19	ND a	1.70 A ± 0.10	9.16 ^A^ ± 0.40
Methionine		10.16 a ± 2.67	12.77AB ± 5.42	15.06 ^A^ ± 1.97	11.94 a ± 1.68	20.41 B ± 1.37	27.94 ^B^ ± 7.65	7.15 a ± 0.27	18.49AB ± 1.57	13.25 ^A^ ± 0.75	40.61 b ± 4.55	18.62 AB ± 2.56	15.71 ^A^ ± 0.95	8.64 a ± 0.45	11.01 A ± 2.27	15.37 ^A^ ± 2.50
Phenylalanine		3.35 a ± 3.21	26.32 A ± 3.55	41.65 ^B^ ± 4.03	33.27 b ± 1.99	47.16 B ± 3.54	67.74 ^D^ ± 1.36	42.66 c ± 3.49	44.83 B ± 1.24	34.28 ^AB^ ± 1.73	46.10 c ± 2.32	49.21 B ± 1.06	58.13 ^C^ ± 4.38	ND a	24.58 A ± 3.21	27.20 ^A^ ± 1.41
Proline		24.40 a ± 5.54	37.74 B ± 4.50	38.01 ^A^ ± 3.00	66.40 c ± 5.45	69.31 C ± 2.71	96.57 ^C^ ± 3.87	48.73 b ± 5.14	68.80 C ± 0.44	79.51 ^B^ ± 0.72	80.00 d ± 0.96	75.19 C ± 4.10	72.92 ^B^ ± 2.86	22.39 a ± 1.74	23.88 A ± 3.25	31.45 ^A^ ± 1.09
Serine		ND a	ND A	ND ^A^	11.54 b ± 1.85	22.95 D ± 1.42	24.81 ^D^ ± 2.01	ND a	6.51 B ± 0.42	5.52 ^B^ ± 0.30	ND a	11.81 C ± 1.88	20.18 ^C^ ± 1.82	ND a	ND A	ND ^A^
Threonine		ND	ND	ND	ND	ND	ND	ND	ND	ND	ND	ND	ND	ND	ND	ND
Tryptophan		ND a	ND A	20.89 ^A^ ± 2.64	ND a	20.08 B ± 1.70	21.71 ^A^ ± 0.80	38.43 c ± 1.24	20.86 B ± 0.90	22.57 ^A^ ± 2.74	33.38 b ± 2.84	28.78 C ± 0.90	21.96 ^A^ ± 1.19	ND a	19.97 B ± 1.40	21.94 ^A^ ± 1.32
Tyrosine		ND a	ND A	ND ^A^	42.05 b ± 1.95	62.61 B ± 5.10	38.33 ^B^ ± 2.60	ND a	ND A	ND ^A^	86.90 c ± 4.87	77.11 C ± 1.48	52.00 ^C^ ± 2.04	3.65 a ± 0.35	ND A	ND ^A^
Valine		30.04 a ± 4.34	36.32 AB ± 5.44	34.27 ^A^ ± 4.22	47.32 b ± 2.01	100.48 D ± 7.25	145.05 ^C^ ± 5.90	23.78 a ± 3.55	44.09 B ± 3.40	41.63 ^A^ ± 2.95	63.02 c ± 2.49	67.03 C ± 1.57	76.75 ^B^ ± 4.67	22.83 a ± 3.19	30.82 A ± 1.27	37.89 ^A^ ± 1.73
Cystine		ND	ND	ND	ND	ND	ND	ND	ND	ND	ND	ND	ND	ND	ND	ND
Taurine		13.58 a ± 1.14	29.50 B ± 4.89	49.87 ^D^ ± 0.93	10.50 a ± 0.69	12.89 A ± 1.66	15.85 ^A^ ± 1.45	11.10 a ± 2.03	12.62 A ± 1.43	15.55 ^A^ ± 0.54	38.70 c ± 1.66	35.63 B ± 2.80	30.00 ^B^ ± 1.38	18.34 b ± 1.09	32.91 B ± 2.18	38.78 ^C^ ± 3.58
Citrulline		21.28 a ± 2.22	25.93 A ± 5.96	29.73 ^B^ ± 3.91	22.76 a ± 2.26	28.26 A ± 2.89	26.86 ^AB^ ± 1.42	29.60 b ± 1.17	25.55 A ± 1.11	22.59 ^A^ ± 0.41	25.14 ab ± 1.66	23.40 A ± 0.87	21.91 ^A^ ± 1.55	22.80 a ± 1.07	23.33 A ± 0.78	27.07 ^AB^ ± 3.11
Sarcosine		32.78 b ± 8.88	ND A	ND ^A^	ND a	ND A	ND ^A^	45.32 c ± 2.96	41.64 B ± 2.63	33.26 ^B^ ± 2.64	36.28 bc ± 2.83	ND A	ND ^A^	37.52 bc ± 1.67	ND A	ND ^A^
Ornithine		ND a	33.36 A ± 3.71	37.72 ^A^ ± 3.39	46.03 b ± 1.13	54.96 B ± 3.98	77.60 ^D^ ± 3.38	103.43 d ± 3.13	54.50 B ± 3.64	46.44 ^B^ ± 3.33	88.20 c ± 3.21	57.67 B ± 4.18	55.78 ^C^ ± 2.42	ND a	34.39 A ± 0.72	36.28 ^A^ ± 2.74
Total free amino acids (TFAA)		447.94 a ± 16.08	574.22 A ± 9.36	800.92 ^B^ ± 28.63	595.99 b ± 20.13	1255.57 D ± 30.56	1555.83 ^D^ ± 33.50	775.69 c ± 6.67	881.57 B ± 19.30	1142.14 ^C^ ± 15.02	1083.51d ± 25.40	1152.98C ± 38.18	1596.64 ^D^ ± 53.44	409.91 a ± 7.57	520.53 A ± 15.38	611.02 ^A^ ± 20.54

ND—not detected; means ± standard deviations; a–d—means with different letters in line for 1 week of ripening are significantly different (*p* < 0.05, *n* = 6); A–D—means with different letters in line for 3 weeks of ripening are significantly different (*p* < 0.05, *n* = 6); ^A–D^—means with different superscript letters in line for 5 weeks of ripening are significantly different (*p* < 0.05, *n* = 6).

**Table 2:** Concentration of biogenic amines (BAs) in Dutch-type cheese models during ripening (mg kg^−1^).

Added		Control			*Lb. rhamnosus* 489			*Lb. delbrueckii* 490			*Lb. acidophilus* 2499			*Lb. casei* 2639		
Biogenic Amines	Time of Ripening (Week)	1	3	5	1	3	5	1	3	5	1	3	5	1	3	5
Agmatine		ND	ND	ND	ND	ND	ND	ND	ND	ND	ND	ND	ND	ND	ND	ND
Phenylethylamine		ND	ND	ND	ND	ND	ND	ND	ND	ND	ND	ND	ND	ND	ND	ND
Histamine		17.69 a ± 0.72	22.33 A ± 2.25	40.98 ^A^ ± 4.93	36.21 c ± 1.61	58.46 D ± 3.05	71.64 ^B^ ± 3.31	57.07 d ± 1.66	51.83 C ± 2.14	41.05 ^A^ ± 2.26	53.25 d ± 1.68	51.50 C ± 1.73	46.61 ^A^ ± 3.26	27.03 b ± 1.58	32.16 B ± 2.12	45.51 ^A^ ± 2.16
Cadaverine		0.64 a ± 0.09	0.78 AB ± 0.10	4.62 ^B^ ± 0.91	0.67 a ± 0.06	0.69 A ± 0.01	0.69 ^A^ ± 0.00	4.36 b ± 0.30	1.07 C ± 0.13	0.41 ^A^ ± 0.10	5.46 c ± 0.34	0.87 ABC ± 0.08	0.54 ^A^ ± 0.06	0.59 a ± 0.07	0.92 BC ± 0.04	0.89 ^A^ ± 0.02
Putrescine		0.32 b ± 0.10	0.38 B ± 0.30	3.05 ^B^ ± 0.28	0.21 b ± 0.00	ND A	ND ^A^	ND a	ND A	4.62 ^C^ ± 1.06	1.17 c ± 0.10	0.27 AB ± 0.08	ND ^A^	ND a	0.64 B ± 0.04	0.46 ^A^ ± 0.02
Spermidine		1.62 d ± 0.12	1.17 D ± 0.23	0.48 ^A^ ± 0.03	0.29 b ± 0.01	0.40 B ± 0.04	0.37 ^A^ ± 0.01	ND a	ND A	0.92 ^B^ ± 0.11	ND a	0.76 C ± 0.10	1.19 ^B^ ± 0.31	0.56 c ± 0.05	1.08 D ± 0.04	1.03 ^B^ ± 0.01
Spermine		1.60 b ± 0.10	0.31 BC ± 0.10	ND ^A^	0.29 a ± 0.10	0.23 B ± 0.00	ND ^A^	3.26 c ± 0.26	ND A	ND ^A^	1.30 b ± 0.04	0.37 C ± 0.04	0.34 ^B^ ± 0.05	ND a	0.21 B ± 0.01	0.56 ^C^ ± 0.06
Tryptamine		2.49 b ± 0.36	3.88 C ± 1.08	ND ^A^	6.95 c ± 0.45	4.85 C ± 0.29	4.70 ^C^ ± 0.16	ND a	ND A	1.72 ^B^ ± 0.35	ND a	1.88 B ± 0.07	ND ^A^	ND a	5.03 C ± 0.06	ND ^A^
Tyramine		1.21 b ± 0.06	1.32 AB ± 0.37	4.29 ^B^ ± 0.31	0.43 a ± 0.04	ND A	ND ^A^	2.34 c ± 0.11	6.77 C ± 1.26	ND ^A^	4.80 d ± 0.25	1.87 B ± 0.08	ND ^A^	0.42 a ± 0.03	ND A	ND ^A^
Total BA		25.56 a ± 1.13	30.17 A ± 3.19	53.41 ^A^ ± 4.78	45.05 b ± 2.21	64.64 C ± 3.18	77.41 ^B^ ± 3.41	67.03 c ± 1.22	59.67 C ± 2.70	48.72 ^A^ ± 3.16	65.98 c ± 1.12	57.52 C ± 1.73	48.68 ^A^ ± 3.09	28.61 a ± 1.63	40.04 B ± 2.13	48.45 ^A^ ± 2.10

ND—not detected; means ± standard deviations; a–d—means with different letters in line for 1 week of ripening are significantly different (*p* < 0.05, *n* = 6); A–D—means with different letters in line for 3 weeks of ripening are significantly different *(p* < 0.05, *n* = 6); ^A–D^—means with different superscript letters in line for 5 weeks of ripening are significantly different (*p* < 0.05, *n* = 6).

**Table 3:** Concentration of bioactive peptides (BP) in Dutch-type cheese models during ripening (mg kg^−1^).

Added		Control			*Lb. rhamnosus* 489			*Lb. delbrueckii* 490			*Lb. acidophilus* 2499			*Lb. casei* 2639		
Bioactive Peptides	Time of Ripening (Week)	1	3	5	1	3	5	1	3	5	1	3	5	1	3	5
Anserine		66.33 b ± 3.79	85.17 A ± 2.98	79.29 ^A^ ± 2.62	71.29 b ± 0.96	114.97 B ± 5.44	99.20 ^B^ ± 2.54	ND a	101.76 B ± 7.54	92.96 ^AB^ ± 10.09	93.04 d ± 3.12	114.34 B ± 4.86	119.02 ^C^ ± 2.17	83.55 c ± 3.57	82.55 A ± 7.13	80.64 ^A^ ± 3.56
L-carnosine		8.37 b ± 1.07	ND A	ND ^A^	33.08 c ± 2.80	32.53 C ± 2.36	3.74 ^B^ ± 0.51	ND a	ND A	ND ^A^	ND a	8.74 B ± 0.75	17.09 ^C^ ± 0.94	ND a	ND A	ND ^A^
Total BP		74.70 b ± 4.87	85.17 AB ± 2.98	79.29 ^A^ ± 2.62	104.37 e ± 2.28	147.50 D ± 7.78	102.94 ^B^ ± 3.00	ND a	101.76 B ± 7.54	92.96 ^AB^ ± 10.09	93.04 d ± 3.12	123.07 C ± 5.39	136.11 ^C^ ± 2.67	83.55 c ± 3.57	82.55 A ± 7.13	80.64 ^A^ ± 3.56

ND—not detected; means ± standard deviations; a–d—means with different letters in line for 1 week of ripening are significantly different (*p* < 0.05, *n* = 6); A–D—means with different letters in line for 3 weeks of ripening are significantly different (*p* < 0.05, *n* = 6); ^A–D^—means with different superscript letters in line for 5 weeks of ripening are significantly different (*p* < 0.05, *n* = 6).
